# Department of Surgery

**Published:** 1900-11

**Authors:** W. E. A. Wyman, J. A. Sloan, Robert Jay

**Affiliations:** Burlington, Wis.; San Francisco, Cal.; West Liberty, Ia.


### DEPARTMENT OF SURGERY.

By W. E. A. Wyman, M.D.V., V.S.,
BURLINGTON, WIS.

J. A. Sloan, B.Sc., M.D.V.,
SAN FRANCISCO, CAL.

Robert Jay, M.D.V.,

WEST LIBERTY, IA.

### Hoof Diseases.

(Continued from page 630.)

While holding the hoof in both hands in the manner described a full view of the sole, frog, and bars is obtained. Is the horn dry and brittle or moist and soft? Is the frog well developed and smooth, especially the middle lacuna, as it should be? Let us suppose the sole is removed. First the sole: Is its concavity normal? Is it a drop sole, or is it a contracted sole characterized by excessive concavity? Is the white line in full union with the sole, or are separations present? Next the bars. Is their course correct? Are both alike? Are bar-cracks present? Along the bearing surface of the shoe and in the angle formed by the bars and the wall it is well to remove a thin layer of the horn, simply to see its color at these parts. Here bruises, corns, suppurations are recognized by the reddish, bluish, brownish, specked or black color of the horn, and a peculiar yellowish and red diffused state of the horn suggests a hemorrhagic or serous pododermatitis, as we shall see later. The frog is examined for foreign bodies, as nails, etc., atrophy, thrush, etc.

Palpation of the hoof with the pincers or hammer requires practice in order to avoid making diagnostic errors. Even a perfectly healthy hoof when tested injudiciously with hammer or pincers will exhibit pain, for instance, when pared recently, and the pincers applied with too much pressure. The question naturally arises, How much pressure may be applied to get safe results? Compress the handles of the pincers with a short movement, applying just enough force to bend the horn of the part pressed upon a little. This rule is for the beginner. As he proceeds he will soon learn to size up a hoof and the amount of pressure it may stand to give reliable results on palpation. Whenever the hammer or pincers cause pain by their application the animal shows it by a more or less vigorous reflex movement in the triceps extensor of the foreleg, and in the hind leg, in the muscle of the croup and thigh mainly. Therefore, do not watch the spot you press upon, but closely observe the muscles alluded to for any short contractions. Another rule worth remembering in testing a hoof with the pincers consists, in starting, in compressing first that portion of the sole, or whatever part one is examining, which is known to be in a healthy state and therefore not in pain. This gives you a chance to acquaint yourself with the normal elasticity of the hoof, and by gradually working the various portions of the hoof with the pincers in that way a painful area is more readily appreciated when struck. Any painful spot after thus being located by palpation may then be further examined with the hoof-knife. On the whole, the writer prefers a percussion hammer to the pincers. In fact, certain diseased states, unless recognized by inspection—for instance, loose wall—can only be diagnosed with the hammer. Nevertheless, the pincers when properly applied give very satisfactory results, and are more adapted for the use of the beginner than the hammer. Test the hoof by applying one jaw upon the outer, the other one upon the inner quarter ; next start one jaw in the angle formed by the bar and wall, going around the sole until you arrive at a spot opposite where you started from, taking a new bite every inch or so, paying especial attention to the nail-holes. Finally, place one jaw up just in front of the anterior commissure of the middle lacuna of the frog, while the other jaw is rested about in the middle of the wall of a quarter. Now move the outer jaw to the middle of the wall of the toe, leaving the other jaw rest upon the frog. Having tested this part, move the outer jaw once more until it arrives opposite its first position—that is, when the outer wall and middle of the frog were compressed first. Lastly, the inner wall and middle of the frog are tested. This last test is first employed in testing the region of the navicular joints.

The heels and plantar cushion are best examined by both inspection and palpation. Inflammatory states of these parts are characterized by a change in form, thus one heel may project more and stand higher than the other one, or both may be out of place under these circumstances. The coronary portion bulges out more or less over the horny wall, often being of a fatty, glistening appearance. The plantar cushion when involved, beside other well-marked symptoms, induces a filling of the hollow of the heels. Palpation of the heels in such states, also the plantar cushions, is accompanied by more or less pain. To palpate the hoof it is not necessary to remove the shoe. Only when pain results from palpation with the pincers or hammer may removal of the shoe become necessary.

As far as the hoof proper is concerned there remains but one more point of special interest, and that is ring-formation, either physiological or pathological, as the case may be. The physiological rings, as a rule, involve the whole hoof, run parallel with the coronet, and only involve the periople and the uppermost layers of the horny capsule, and simply represent physiological increase in the blood-supply or the horn-producing parts of the coronary cushion. They follow a change of food. For instance, a horse out to pasture for six months, suddenly put on an oats diet, will in due time show these rings. Pregnancy, etc., also are concerned in the production of them. Pathological ring-formation usually involves only a part of the hoof; while it runs parallel with the coronet it depends on a dislocation of the papillary body of the coronary cushion, either by retraction upon or compression of those parts.

In conclusion, examination of the coronet, bones, and articulation of the phalanges must be made.

In the coronary region one looks for swelling, which may be simple or purulent inflammations of the skin and subcutis, fistulous tracts of quittors, or suppurating corns (a glaring misnomer). Fulness of the coronet may also depend on a stasis, as inflammation of the podophyllous tissue, which swelling may deceive the beginner, causing him to think of a tendonitis; but the excessive pulsation of the arteries carrying blood to the hoof, with other well-marked symptoms, should dispel any doubts in that direction. Dislocation of the os pedis as seen in serious cases of laminitis permits the coronary region to descend, giving it a sunk-in appearance.

While examining the coronary region an examination of the lateral cartilages should be made. The point of greatest interest is their elasticity, depending on their form, which is as variable as the hoofs they equip. The amount of elasticity lies within wide limits, and practice only can teach when a decreased elasticity is encountered. To test the cartilage the hoof is held in both hands, as described when inspecting the sole. The thumb is placed upon the superior border, and down and inward pressure exerted. An honest test—that is, a reliable test—to perceive the elasticity of the deeper parts the writer is not acquainted with, and the early and deep ossification of the lateral cartilages may be diagnosed by exclusion only. It has been suggested to use the hoof pincers over the ossifying member, and that expression of pain on palpation was suggestive of ossification of the lateral cartilage. The mere fact that such a test only "suggests" the diseased state renders it too uncertain a means to be employed to settle doubtful cases.

The diagnosis of fracture of the os pedis or navicular, or the differential diagnosis, is at times exceedingly difficult, and, as a rule, an exhaustive examination of all parts of the leg must be made and the case diagnosed by exclusion. The most reliable symptom of fracture of the navicular bone so far known to the writer was pointed out to him some years ago by Dr. M. H. McKillip, consisting of a peculiar set-back or slight descent of the anterior face of the second interphalangeal articulation.

The individual examination of the phalangeal articulations and their ligaments is with some of them exceedingly difficult, as reliable information can only be obtained when each joint is examined by itself without the examination exerting its influence at the same time upon the other joints, as it is the case, for instance, in passive rotation of the second interphalangeal articulation without first fixing the first interphalangeal joint. In other words, by simply getting hold of the hoof and rotating it with a view to produce pain would, to be charitable, yield a mixed diagnosis. To properly test for a painful state of the second interphalangeal articulation by positive movements, the first interphalangeal articulation must be fixed. The foot is elevated, the assistant firmly embraces the fetlock, while the os corona and os pedis are forcibly extended. In this position the upper joint is practically fixed, and flexion, extension, and rotation of the second interphalangeal joint may now be practised and reliable results obtained. To test the first interphalangeal articulation the second one must be fixed. The foot is elevated, the fetlock held firmly, and strong flexion of the second interphalangeal articulation practised. In this way the navicular bone is pushed against the os corona so closely as to practically lock the joint. The metacarpo-phalangeal articulation is readily examined both by inspection and palpation. Here passive extension and flexion may also be employed to advantage, while rotation is practically *nil* on account of the anatomical arrangement of the articulation.

Finally, it may be remembered that two main forms of pus are met with. One is thin, gray on the black hoof, or light brown on the non-pigmented hoof; the other is of creamy color and thick. The former is the product of a superficial inflammation; the latter the result of a deep and more serious inflammatory state.

While speaking of the difficulty in examining the hoof in case of fracture of the navicular bone, os pedis, ossification of the lateral cartilage, the writer pointed out the difficulties experienced and the doubtful results obtained by the ordinary means of examination. The employment of the X-ray permits one positively to settle such doubtful points. The employment of the X-ray in the diagnosis of certain pathological states is of necessity but little employed in veterinary surgery, nevertheless it is practical, its application requires but little time (about two minutes or even less), and after the eye has once been trained to appreciate correctly what it perceives, little difficulty is experienced in employing it to advantage. The reason that it cannot be employed largely is simply that it takes too much of the almighty dollar to establish an X-ray apparatus. In the following pathological states it may be employed with advantage: Ossification of the lateral cartilage, fractures and fissures of any of the phalangeal bones, nail pricks, ringbones, while it is to be regretted that chronic navicular arthritis, as a rule, is not detectable by the Röntgen ray.

---

The *Revista de medecina y chirurgia Practicas* for July 21st, citing *Progrès Médicale*, says that Drs. Roger and Garnier recently reported to the Paris Society of Biology the following facts on the transmission of tuberculosis by human milk: They found the bacilli of tuberculosis in the milk of a woman who died from miliary tuberculosis seventeen days after childbirth without the existence of any tuberculous lesions in the mammary glands. This milk was taken with aseptic precautions and inoculated into two small Indian rabbits. The first, which had received subcutaneously four cubic centimetres, succumbed after thirty-three days with generalized typical tuberculous lesions; the second, in which only two cubic centimetres were injected, did not die, and when it was killed, ten months later, only simple non-tuberculous cicatrices were found. The infant died after six months with tubercles in the mesenteric glands, the liver, spleen, and kidneys. From these facts it appears that the intestine formed the point of entrance. This case, the author thinks, shows that the milk from a tuberculous woman may contain the bacilli of Koch and transmit tuberculosis.—*New York Medical Journal*, September 1, 1900.

